# The protective impact of education on brain structure and function in Alzheimer’s disease

**DOI:** 10.1186/s12883-021-02445-9

**Published:** 2021-10-30

**Authors:** Wanqiu Zhu, Xiaoshu Li, Xiaohu Li, Haibao Wang, Meiqin Li, Ziwen Gao, Xingqi Wu, Yanghua Tian, Shanshan Zhou, Kai Wang, Yongqiang Yu

**Affiliations:** 1 Department of Radiology, The First Affiliated Hospital of Anhui Medical University, No. 218, Jixi Road, Shushan District, Hefei, 230022 China; 2 Department of Neurology, The First Affiliated Hospital of Anhui Medical University, Hefei, 230022 China

**Keywords:** Alzheimer’s disease, Cognitive reserve, Education, Gray matter volume, Resting-state fMRI

## Abstract

**Background:**

The Cognitive Reserve (CR) theory posits that brains with higher reserve can cope with more cerebral damage to minimize clinical manifestations. The aim of this study was to examine the effect of education (CR proxy) on brain structure and function in Alzheimer’s disease (AD) and amnestic mild cognitive impairment (aMCI) patients and in cognitively healthy elderly (HC) individuals.

**Methods:**

Fifty-seven AD patients, 57 aMCI patients and 48 HCs were included to investigate the relationships between education years and gray matter volume (GMV), regional homogeneity (ReHo) and functional connectivity (FC) in brain regions to show associations with both structure and function. Taking the severity of the disease into account, we further assessed the relationships in AD stratified analyses.

**Results:**

In AD group, the GMV of the dorsal anterior cingulate cortex (dACC) and ReHo in the left inferior temporal cortex (ITC) were inversely associated with education years, after adjustment for age, sex, Mini-Mental State Examination (MMSE), and total intracranial volume or head motion parameters. Seed-based FC analyses revealed that education years were negatively correlated with the FC between the left anterior ITC and left mid frontal cortex as well as right superior frontal cortex and right angular gyrus. Stratified analyses results indicated that this negative relation between education and GMV, ReHo, FC was mainly present in mild AD, which was attenuated in moderate AD and aMCI groups.

**Conclusions:**

Our results support the CR theory, and suggest that CR may be protective against AD related brain pathology at the early stage of clinical dementia. These findings could provide the locus of CR-related functional brain mechanisms and a specific time-window for therapeutic interventions to help AD patients to cope better with the brain pathological damage by increasing CR.

**Supplementary Information:**

The online version contains supplementary material available at 10.1186/s12883-021-02445-9.

## Background

The reserve theory is established to interpret the apparent discrepancy between brain pathology and its clinical manifestations in different individuals. The theory posits that certain life experiences enable some people to withstand the brain pathology more effectively compared with others through enhancing the adaption of neural networks [1, 2]. Since the changes in brain recruitment associated with reserve are a normal response to increased task demands, this definition suggests that cognitive reserve is present in both healthy individuals and those with brain damage [3]. The cognitive/functional brain processes that support CR may already be present before the onset of brain pathologies. Most studies support the reserve hypothesis in neurodegenerative disorders. In Alzheimer’s disease (AD), patients with higher cognitive reserve (CR) can tolerate greater brain pathological damage at a particular degree of cognitive impairment than those with lower CR, such as coping with decreased cerebral ^18^F-fluorodeoxyglucose positron emission tomography (FDG-PET) metabolism [4–8], cortical thinning, or gray matter volume (GMV) atrophy [4, 6, 9–11]; having more severe white matter damage [12, 13]; and also primary AD pathologies such as greater amyloid-beta (Aβ) accumulation and increased tau deposition [14–16].

Reserve is an abstract construct that cannot be measured directly. Several findings demonstrated that education was positively correlated with cognition level and thus influenced the risk of dementia. It is suggested that education should be deemed as the main indirect evaluation index of CR, though other indexes such as occupation or IQ may also be considered [17–19]. Higher educated individuals seem to withstand more severe brain damage. Although it is not a perfect measurement of CR, education has been used widely with diverse populations and in various studies, thus reinforcing the rationale for testing its connection to dementia. A crucial question is whether our measurement of brain changes could be used as CR-related biomarkers of AD pathology. In a recent study, cerebrospinal fluid (CSF) Aβ42, CSF tau, FDG-PET, and structural and functional MRI have been suggested as biomarkers of AD-related pathology [20]. Therefore the measures of AD-related pathology we used here were MRI-based measures of brain structure and function.

To test the CR hypothesis, in the present study, we investigated whether higher education is associated with comparatively lower regional GMV, lower regional homogeneity (ReHo) and decreased functional connectivity (FC) at a given level of global cognitive function in AD. We hypothesized that despite more severe brain structural and functional damage, highly educated elderly AD individuals could preserve cognitive ability comparable to poorly educated patients.

## Methods

### Participants

The total sample in this study was composed of 162 participants (57 AD patients, 57 aMCI patients, and 48 HCs). The AD and aMCI patients were recruited from the Dysmnesia Outpatient Department at the first Affiliated Hospital of Anhui Medical University in Hefei, Anhui Province, China between 2017 and 2019. The HCs were recruited from the local community or were the spouses of the patients in the study. The diagnosis of AD in the study was established according to the National Institute of Neurological and Communicative Disorders and Stroke-Alzheimer’s Disease and Related Disorders Association (NINCDS-ADRDA) criteria of “probable” or “possible” AD [21, 22] and that patients showed medial temporal atrophy on structural magnetic resonance imaging [22, 23]. The diagnosis of aMCI was established according to the Petersen criteria [24, 25] and patients had a Clinical Dementia Rating (CDR) score of 0.5. All aMCI subjects had memory complaints confirmed by neuropsychological testing, while absence of dementia. 32 aMCI patients (single domain) exhibit memory problems only, whereas 25 aMCI patients (multiple domain) also have impaired visuospatial ability. The criteria for HC comprised the following: (1) normal general physical status; (2) normal global cognitive function as measured by the MMSE (score range between 24 and 30); (3) CDR memory score of 0; and (4) without memory complaints. Exclusion criteria for all participants included: history of other neurological or psychiatric diseases or head injury with loss of consciousness, use of sedative drugs in the last 24 h before the neuropsychological assessment, drug or alcohol addiction, prior chronic exposure to neurotoxic substances.

### Neuropsychological assessment

All participates were right-handed and underwent clinical evaluation and neuropsychological assessment. Educational attainment was assessed in terms of the highest year of schooling completed. The following neuropsychological test battery was administered to each subject for the purpose of establishing a clinical diagnosis [26]. (i) General cognitive function was assessed by the MMSE [27] and CDR score [28]. The severity of dementia was assessed by a neuropsychologist using both the CDR scale and the MMSE, defining the initial severity level of AD, which corresponded to mild (CDR 1), moderate (CDR 2), or severe dementia (CDR 3) [15]. (ii) The Chinese version of the auditory verbal learning test (AVLT) was used to evaluate episodic memory ability [29]. (iii) The verbal fluency test (VFT) was used to assess semantic memory ability [30]. (iv) The Digit Span Forward and Backward tests were used to evaluate working memory ability [31]. All participants gave their written informed consent and the study was approved by the Medical Research Ethics Committee of the first Affiliated Hospital of Anhui Medical University.

### Neuroimaging protocol acquisition

All participants were scanned on a General Electric Discovery MR750w 3.0 T scanner (General Electric, Waukesha, WI, USA) with a 24-channel head coil. MRI examinations were performed within 2 days after the completion of the cognitive tests. The MR imaging protocol included routine T2-weighted and fluid-attenuated inversion recovery (FLAIR) images, three-dimensional (3D) high-resolution T1-weighted structure images, and resting-state functional MRI (rs-fMRI) images. Routine T2-weighted and FLAIR images were used to exclude subjects with organic brain diseases. 3D high-resolution T1-weighted structure images were acquired using the brain volume (BRAVO) sequence (TR = 8.5 ms; TE = 3.2 ms; TI = 450 ms; flip angle = 12°; 188 slices; slice thickness = 1 mm; no gap; FOV = 256 × 256 mm^2^; matrix = 256 × 256). The rs-fMRI scans were performed with an echo planar imaging sequence (TR = 2000 ms; TE = 30 ms; flip angle = 90°; slice thickness = 3 mm; slice gap = 1 mm; FOV = 220× 220 mm^2^; matrix = 64 × 64). A custom-built head coil cushion and earplugs were used to minimize head motion and dampen scanner noise. During the scans, subjects were instructed to keep their eyes closed, relax, and move as little as possible while not falling asleep.

### White matter hyperintensity evaluation

The severity of white matter hyperintensity (WMH) was assessed visually on axial FLAIR sequences according to the modified Fazekas scale [32, 33]. This scale divided WMH into periventricular and deep categories. Periventricular WMH was graded according to the following patterns: 0 = absent, 1 = caps or a pencil-thin lining, 2 = a smooth halo, and 3 = irregular WMH extending into the deep white matter. Deep WMH was graded according to the following patterns: 0 = absent or single punctate foci, 1 = multiple punctate foci, 2 = beginning confluence of foci, and 3 = large fused foci. The total scores were acquired by adding the periventricular and deep WMH scores together. Two radiologists assessed and scored the WMH burden independently.

### Voxel-based Morphometry analysis

The 3D T1-weighted structural images were processed using the VBM8 toolbox (http://dbm.neuro.uni-jena.de/vbm.html) in Statistical Parametric Mapping software (SPM8; http://www.fil.ion.ucl.ac.uk/spm). First, all structural images were visually inspected to screen for artifacts or gross anatomical abnormalities, after which they were segmented into gray matter, white matter, and cerebrospinal fluid using the standard segmentation model. After an initial affine registration of the gray matter concentration map into Montreal Neurological Institute (MNI) space, the gray matter concentration images were nonlinearly warped using the diffeomorphic anatomical registration through exponential Lie algebra (DARTEL) technique and then resampled to a voxel size of 1.5 mm × 1.5 mm × 1.5 mm [34]. The gray matter volume (GMV) map was obtained by multiplying the gray matter concentration map by the non-linear determinants that were derived from the spatial normalization step. Finally, the resultant GMV images were smoothed with a 8-mm full-width at half-maximum Gaussian kernel.

### fMRI data preprocessing

Resting-state BOLD data were preprocessed using SPM and Data Processing & Analysis for Brain Imaging (DPABI, http://rfmri.org/dpabi). The first ten volumes for each participant were discarded to allow the signal to reach equilibrium and the participants to adapt to the scanning noise [35]. The 175 remaining volumes were corrected for the acquisition time delay between slices. Then, realignment was performed to correct the motion between time points. Head motion parameters were computed by estimating the translation in each direction and the angular rotation on each axis for each volume. All participants’ BOLD data were within the defined motion thresholds (i.e., translational or rotational motion parameters less than 3.0 mm or 3.0°). We also calculated frame-wise displacement (FD), which indexes the volume-to-volume changes in head position. Several nuisance covariates (the linear drift, the estimated motion parameters based on the Friston-24 model, the spike volumes with FD > 0.5, the white matter signal, and the cerebrospinal fluid signal) were regressed out from the data. The datasets were then band-pass filtered using a frequency range of 0.01 to 0.08 Hz. In the normalization step, individual structural images were firstly co-registered with the mean functional image, and then the transformed structural images were segmented and normalized to the MNI space using the DARTEL technique. Finally, each filtered functional volume was spatially normalized to MNI space using the deformation parameters estimated during the above step and resampled into a 3-mm cubic voxel.

### ReHo analysis

ReHo assesses local intrinsic FC, being defined as the Kendall’s coefficient of concordance (KCC) for the time series of a given voxel with those of its 26 nearest neighboring voxels [36]. The KCC can be computed by the following formula:

$$W=\frac{\sum {(Ri)}^2-n{\left(\overline{R}\right)}^2}{\left(1/12\right){K}^2\left({n}^3-n\right)}$$

where *W* is the KCC among given voxels, ranging from 0 to 1; *R*_*i*_ is the sum rank of the i^th^ time point; $\overline{R}=\left[\left(n+1\right)K\right]/2$ is the mean of *R*_*i*_; *K* is the number of time series within a measured cluster (*K* = 27, one given voxel plus its 26 neighbors), and n is the number of ranks (*n* = 175). Then, we normalized the ReHo of each voxel by dividing it by the mean ReHo value of the whole brain. Finally, each ReHo map was spatially smoothed with a Gaussian kernel of 6 mm × 6 mm × 6 mm full width at half maximum.

### Resting-state FC (rsFC) analyses

Prior to the rsFC analysis, the preprocessed data were additionally smoothed with a Gaussian kernel of 6 mm × 6 mm × 6 mm full width at half maximum after spatial normalization. The rsFC was examined with a seed-based correlation approach. For the purpose of FC analyses, the regions showing a significant correlation with years of education from both the GMV and ReHo analyses were used as seeds. For each individual data set, Pearson’s correlation coefficients between the mean time series of each seed regions of interest (ROI) and time series of each voxel in the rest of the brain were computed and converted to z-values using Fisher’s r-to-z transformation to improve the normality.

### Statistical analysis

Demographics and clinical characteristics [age, years of education, MMSE score, total intracranial volume, Frame-wise displacement] were compared in AD, aMCI, and HC groups using one-way analysis of variance (ANOVA). Group difference in sex was tested by using Pearson’s Chi-square tests. Each cognitive composite score was computed by converting raw scores on each component of neuropsychological tests to z scores, using the mean and SD for all participants in the study, and averaging the z scores to yield the composite score.

For VBM analyses, voxel wise correlations were computed between education years and the GMV images using the multiple regression analysis of SPM8 in the AD, aMCI, and HC groups, respectively. The same analysis was performed for ReHo and seed-based rsFC. All analyses were controlled for age, sex, MMSE, and total intracranial volume (TIV) or head motion parameters. Considering that the cerebrovascular load may affect the mechanism of structural and functional connectivity of CR, fazekas score was added as a new covariate within the model. Multiple comparisons were corrected using a voxel-level false discovery rate (FDR) method with a significance level of *P* < 0.05. In addition, clusters surviving *P* < 0.05 corrected for family-wise error (FWE) were also considered significant. The regions that showed significant correlations were then considered ROI and the mean GMV and ReHo values in these regions were extracted for the subsequent linear regression analysis.

Given the severity of the dementia symptoms in AD, we further assessed the relationships between years of education and ROI-based neuroimaging variables (GMV, ReHo, and rsFC) in stratified analyses controlling for age, sex, MMSE, and TIV or head motion parameters. Between-group analyses were performed to investigate group difference of ROI-based neuroimaging variables (GMV, ReHo, and rsFC). All *p* values were two-tailed; *P* < 0.05 was considered to be statistically significant. Finally, the Pearson correlation analyses were used to identify correlations between ROI-based neuroimaging variables (GMV, ReHo, and rsFC) with clinical cognitive function scores. All these statistical analyses were performed by using the SPSS 23.0 software package (SPSS, Chicago, IL, USA). All *p* values were two-tailed; *P* < 0.05 was considered to be statistically significant.

## Results

### Demographic and clinical characteristics

Demographics, clinical characteristics and neuropsychological scores are listed in Table 1. The three groups were well matched in age (one-way ANOVA, *F* = 0.491, *P* = 0.613), sex (Chi-square test, χ2 = 0.878, *P* = 0.645), and frame-wise displacement (one-way ANOVA, F = 0.768, *P* = 0.466). There were significant differences in MMSE score (one-way ANOVA, F = 255.546, *P* < 0.001), years of education (one-way ANOVA, F = 18.805, P < 0.001), and TIV (one-way ANOVA, F = 3.884, *P* = 0.023).

**Table 1:** Demographics, clinical characteristics and neuropsychological assessment of the study sample

Characteristics	AD (*n* = 57)	aMCI (n = 57)	HC (*n* = 48)	*p* Value
Women (%)	31 (54)	35 (61)	30(63)	0.645^b^
Age (years)	67.74 (8.28)	66.33 (7.47)	66.75 (7.41)	0.613^a^
Years of education	6.14 (5.38)	9.53 (4.71)	11.69(3.48)	<0.001^a^
MMSE score	15.28 (5.22)	26.35 (1.58)	28.60(1.11)	<0.001^a^
Clinical Dementia Rating Scale				
1 (%)	21 (37)			
2 (%)	22 (39)			
3 (%)	14 (25)			
TIV (cm^3^)	1314.27 (109.09)	1352.93 (99.70)	1368.67 (103.03)	0.023^a^
Frame-wise displacement (mm)	0.21(0.14)	0.22(0.16)	0.18(0.11)	0.466^a^
Fazekas score	2(1–3)	1(0.5–2.5)	1(1–2)	0.229^c^
**neuropsychological tests**				
AVLT (immediate)	2.01(1.62)	5.89(2)	9.04(1.76)	<0.001^a^
AVLT (delay)	0(0–0)	4.46(3.08)	10.13(2.77)	<0.001^c^
AVLT (recognize)	10(6–12)	12(11–14)	14(13.5–15)	<0.001^c^
VFT (Animals)	9(7–10)	13(10.5–14.5)	18.13(3.93)	<0.001^c^
DSpF	5(4–6)	7(6–8)	8(7–8)	<0.001^c^
DSpB	3(2–3)	4(3–5)	5(4–6)	<0.001^c^
**cognitive functionscore**				
Episodic memory^d^	−2.70(1.65)	0.27(1.58)	2.88(1.22)	<0.001^a^
Semantic memory^d^	−0.85(0.63)	0.04(− 0.43–0.32)	1.0(0.74)	<0.001^c^
Working memory^d^	−1.48(1.63)	0.29(1.24)	1.42(1.32)	<0.001^a^

Datas with normal distribution are expressed as mean (SD) or as percentage

Datas with non-normal distribution are expressed as median (interquartile range)

^a^Group difference was tested by using one-way analysis of variance (ANOVA)

^b^Group difference was tested by using Pearson’s Chi-square test

^c^Group difference was tested by using Kruskal-Wallis test

^d^Each cognitive composite score was computed by converting raw scores on each component of neuropsychological tests to z scores, using the mean and SD for all participants in the study, and averaging the z scores to yield the composite score

Abbreviations: *AD* Alzheimer’s disease; *aMCI* amnestic mild cognitive impairment;, healthy control; *MMSE* Mini-Mental State Examination; *TIV* total intracranial volume; *SD* standard deviations; *AVLT* auditory verbal learning test; *VFT* verbal fluency test; *DSpF* Digit Span Forward; *DSpB* Digit Span Backward

### Correlations between GMV and education years

**Fig. 1:**
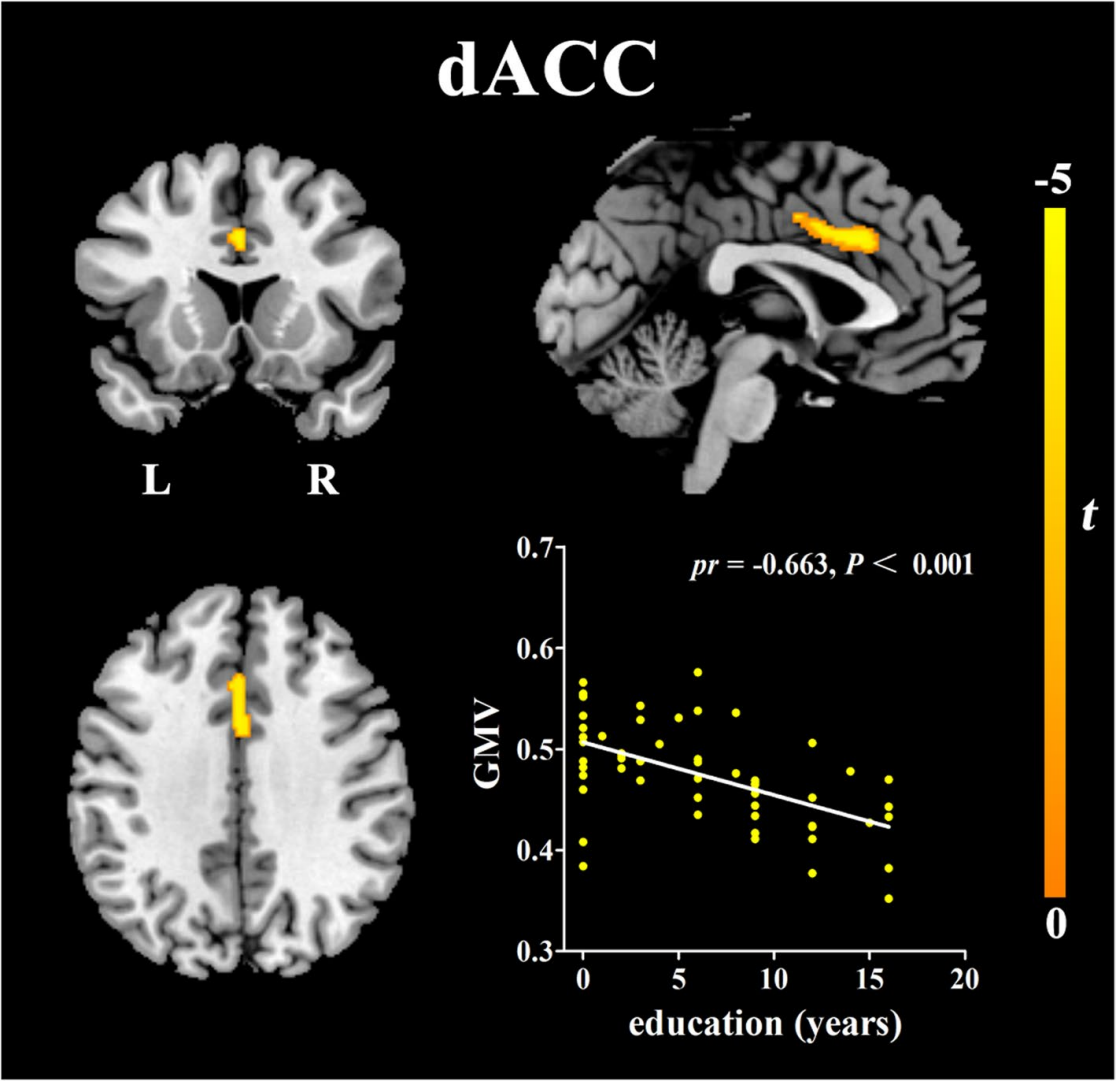
Results of the voxel-wise multiple regression between years of education and gray matter volume after adjustment for age, sex, MMSE and TIV (*P*<0.05, FDR corrected). Details of the peaks are given in Table 2. Abbreviations: dACC, dorsal anterior cingulate cortex; GMV, gray matter volume; L, left; R, right

Controlling for age, sex, MMSE, and TIV, significant negative correlations were observed between education years and the GMV of the dorsal anterior cingulate cortex (dACC) in AD patients (see Fig. 1, Table 2.). No significant negative correlations were observed in aMCI patients or HCs, and no positive correlations were observed in any of the groups. After adding the fazekas score as the new covariate, the similar results were obtained (see Fig. S1, Table S1.).

**Table 2:** Results of voxel-based regression analyses between neuroimaging parameters and years of education

Region	Number of voxels	*t*-score	MNI coordinates		
			x	y	z
**Negative correlations between years of education and GMV**					
dorsal anterior cingulate cortex	291	−5.25	0	20	33
**Negative correlations between years of education and ReHo**					
left anterior part of inferior temporal cortex	65	−6	−57	− 12	−33
left posterior part of inferior temporal cortex	59	−4.69	−51	−30	−21
**Negative correlations between years of education and left aITC rsFC**					
right angular gyrus	32	−4.79	45	−66	42
left mid frontal cortex	27	−5.57	−39	15	57
right superior frontal cortex	16	−4.95	24	27	54

Structural MR (GMV): Adjusted for age, sex, MMSE, and TIV

Resting-state fMRI (ReHo and rsFC): Adjusted for age, sex, MMSE, and head motion parameters

Coordinates (x,y,z) are given in MNI standard space. All regions listed are statistically significant at *p*<0.05 level (FDR corrected)

Abbreviations: *GMV* gray matter volume; *ReHo* Regional Homogeneity; *rsFC* resting-state functional connectivity; *MNI* Montreal Neurological Institute; *aITC* anterior part of inferior temporal cortex; *MMSE* Mini-Mental State Examination; *TIV* total intracranial volume

### Correlations between ReHo and education years

**Fig. 2:**
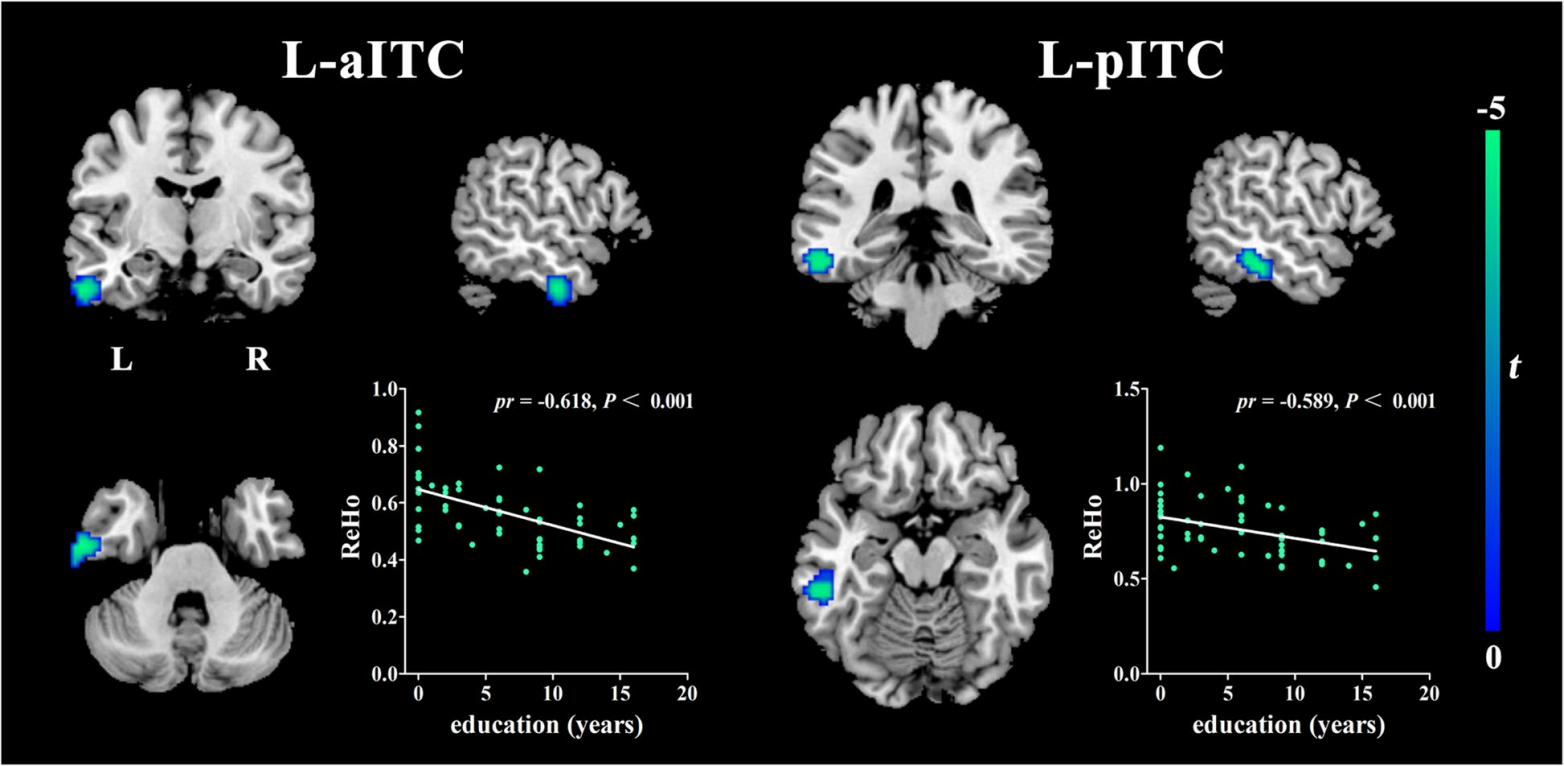
Results of the voxel-wise multiple regression between years of education and mean ReHo values after adjustment for age, sex, MMSE and head motion parameters(*P*<0.05, FDR corrected). Details of the peaks are given in Table 2. Abbreviations: ReHo, Regional Homogeneity; aITC, anterior part of inferior temporal cortex; pITC, posterior part of inferior temporal cortex; L, left; R, right

Controlling for age, sex, MMSE, and head motion parameters, education years were significantly negatively correlated with the ReHo values of the left inferior temporal cortex (ITC) in AD patients (see Fig. 2, Table 2.). No significant negative correlations were observed in aMCI patients or HCs, and no positive correlations were observed in any of the groups. The similar results were also observed after adding the fazekas score as the new covariate (see Fig. S2, Table S1.).

.

### Correlations between seed-based FC and education years

**Fig. 3:**
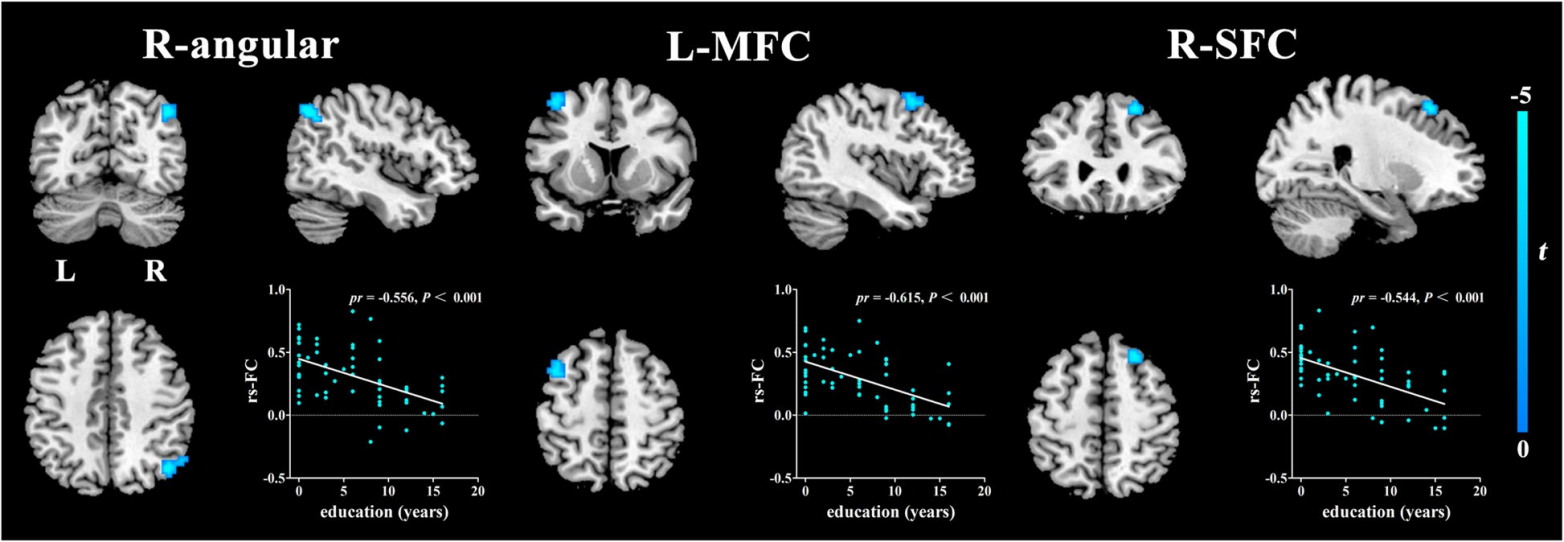
Results of the voxel-wise multiple regression between years of education and left anterior part of inferior temporal cortex (MNI coordinate: − 57,-12,-33) functional connectivity after adjustment for age, sex, MMSE and head motion parameters(*P*<0.05, FDR corrected). Details of the peaks are given in Table 2. Abbreviations: MFC, mid frontal cortex; SFC, superior frontal cortex; rs-FC, resting-state functional connectivity; L, left; R, right

The dACC and ITC were then used as seeds for the whole brain connectivity analyses. Thus we obtained the FC maps and the FC maps were then correlated with education years. Controlling for age, sex, MMSE, and head motion parameters, significant negative correlations were observed between education years and the connectivity of the left anterior ITC (aITC) with the left mid frontal cortex, as well with the right superior frontal cortex and right angular gyrus in AD patients (see Fig. 3, Table 2.). No significant negative correlations were observed in the dACC and posterior ITC (pITC) based FC and no positive correlations were found. The similar results were found after adding the fazekas score as the new covariate (see Fig. S3, Table S1.).

### Correlations between the values extracted from ROI and education years in AD stratified analysis

**Fig. 4:**
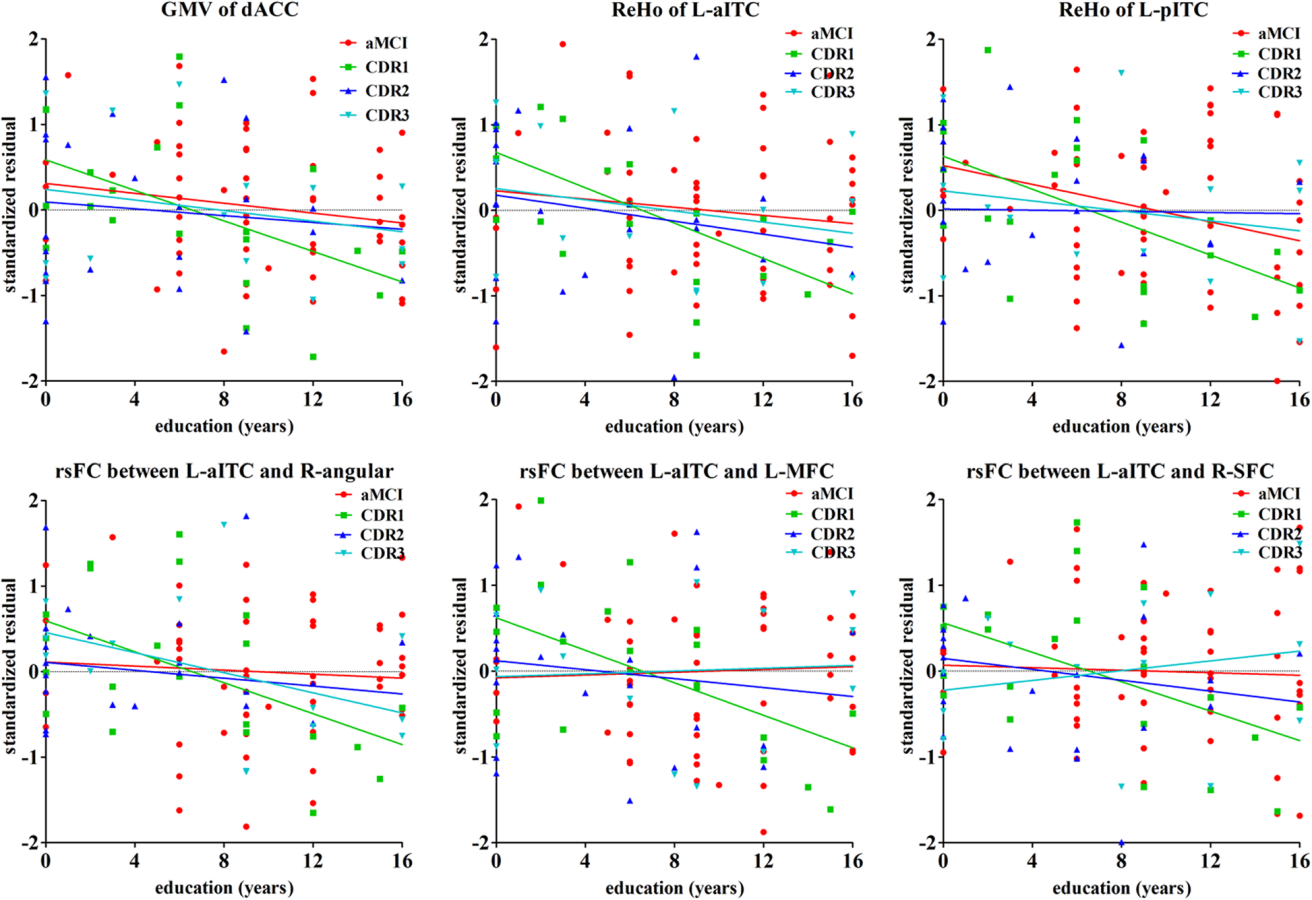
Effect of years of education on AD-related neuroimaging biomarkers, stratified by severity of AD: linear regression analysis. The y axis corresponds to the standardized residuals of GMV, ReHo, and FC values of ROI after adjustment for age, sex, MMSE and TIV or head motion parameters. The x axis corresponds to education years. CDR1 corresponds to a mild form, CDR2 to a moderate form, and CDR3 to a severe form of the disease, aMCI corresponds to preclinical stage of AD. Abbreviations: dACC, dorsal anterior cingulate cortex; GMV, gray matter volume; ReHo, Regional Homogeneity; aITC, anterior part of inferior temporal cortex; pITC, posterior part of inferior temporal cortex; MFC, mid frontal cortex; SFC, superior frontal cortex; rsFC, resting-state functional connectivity; AD, Alzheimer’s disease; aMCI, amnestic mild cognitive impairment; CDR, Clinical Dementia Rating

Table 3 displays the stratified analysis of the correlations between education years and MRI-based biomarkers of AD-related pathology (GMV, ReHo, and FC values of ROI) according to the disease severity (CDR scores). In the mild dementia group, all biomarkers we used were inversely associated with years of education (*P* < 0.01). This inverse relationship was attenuated and not significant in patients with moderate or severe dementia, or in patients with aMCI (see Fig. 4).

**Table 3:** Correlations between years of education and biomarkers of AD-related pathology: linear regression analysis

AD-related pathology biomarkers	Relationships with years of education														
	HC			aMCI			CDR1 (mild dementia)			CDR2 (moderate dementia)			CDR3 (severe dementia)		
	β (SE)	*p*	B	β (SE)	*p*	B	β (SE)	*p*	B	β (SE)	*p*	B	β (SE)	*p*	B
GMV of dACC	−0.001 (0.002)	0.7	−0.062	− 0.003 (0.002)	0.038 *	− 0.258	− 0.007 (0.002)	0.006**	− 0.753	− 0.006 (0.005)	0.233	− 0.569	−0.005 (0.004)	0.239	−0.568
ReHo of left aITC	0.008 (0.005)	0.108	0.261	−0.003 (0.003)	0.372	−0.127	−0.018 (0.004)	0.000***	−0.739	− 0.038 (0.012)	0.006**	−1.485	− 0.007 (0.005)	0.247	− 0.62
ReHo of left pITC	0.003 (0.006)	0.649	0.076	−0.008 (0.004)	0.037 *	−0.284	−0.025 (0.006)	0.002**	−0.831	− 0.003 (0.014)	0.829	− 0.101	−0.011 (0.01)	0.305	−0.586
**left aITC rsFC**															
right angular gyrus	0.002 (0.01)	0.814	0.038	−0.004 (0.009)	0.662	−0.061	−0.034 (0.01)	0.004**	−0.72	− 0.041 (0.026)	0.127	− 0.899	−0.036 (0.012)	0.017 *	−1.043
left mid frontal cortex	0.013 (0.008)	0.098	0.271	0.002 (0.008)	0.763	0.039	−0.031 (0.008)	0.002**	−0.717	−0.035 (0.019)	0.081	−0.873	0.003 (0.011)	0.78	0.103
right superior frontal cortex	0.002 (0.009)	0.835	0.032	−0.002 (0.007)	0.786	−0.038	−0.029 (0.009)	0.007**	−0.658	− 0.05 (0.021)	0.029*	−1.244	0.013 (0.012)	0.317	0.372

Structural MR (GMV): Adjusted for age, sex, MMSE, and TIV

Resting-state fMRI (ReHo and rsFC): Adjusted for age, sex, MMSE, and head motion parameters

Abbreviations: *AD* Alzheimer’s disease; *aMCI* amnestic mild cognitive impairment; *HC* healthy control; *CDR* Clinical Dementia Rating; *SE* standard error; *GMV* gray matter volume; *ReHo* Regional Homogeneity; *rsFC* resting-state functional connectivity; *dACC* dorsal anterior cingulate cortex; *aITC* anterior part of inferior temporal cortex; *pITC* posterior part of inferior temporal cortex; *MMSE* Mini-Mental State Examination; *TIV* total intracranial volume

β stand for unstandardized coefficients, B stand for standardized coefficients (Beta)

*P*< 0.05*, *P*< 0.01**, *P*< 0.001***

### Between-group differences of the values extracted from ROI

**Fig. 5:**
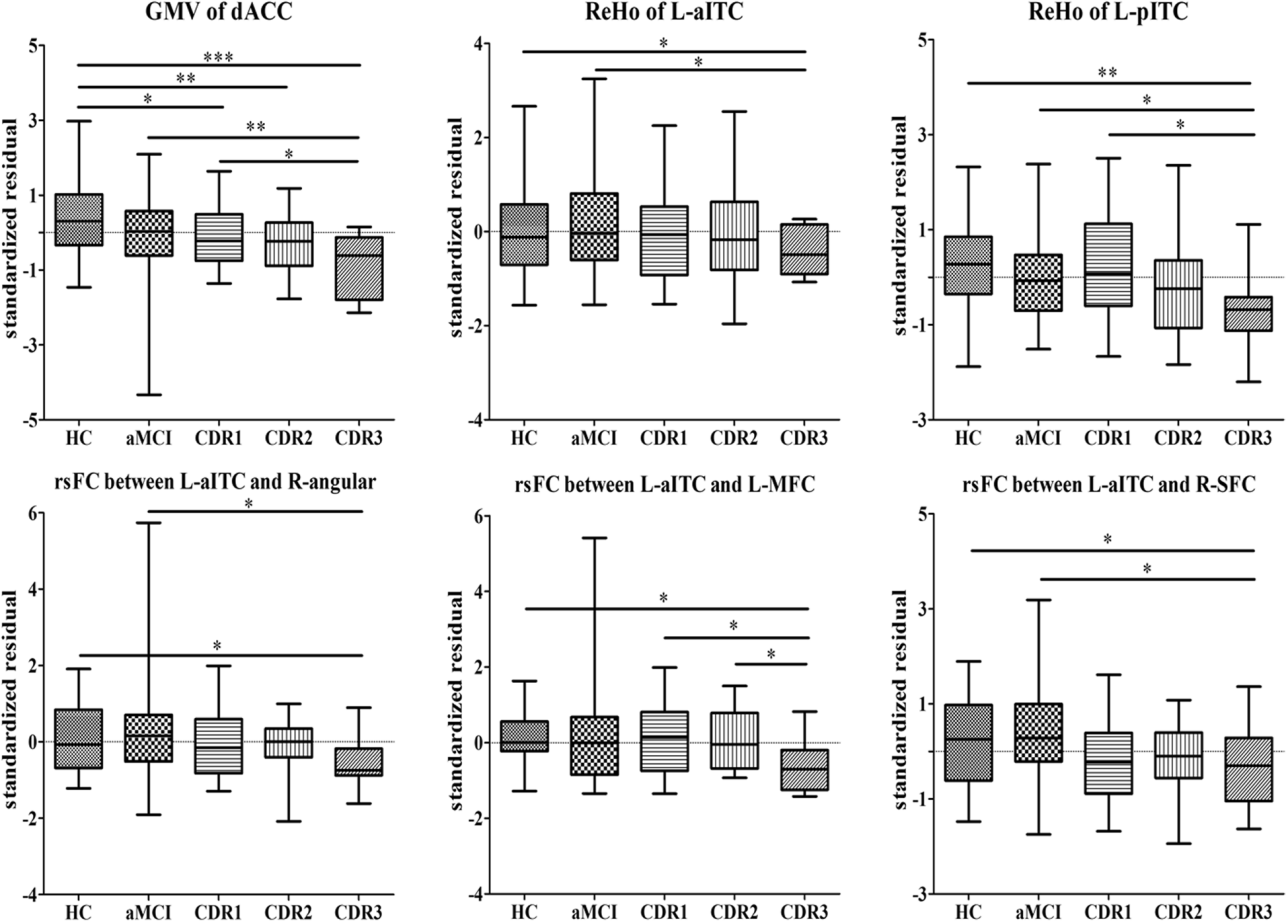
Box plot for the between-group comparison in AD-related neuroimaging biomarkers. The y axis corresponds to the standardized residuals of GMV, ReHo, and FC values of ROI after adjustment for age, sex, and education years. For normally distributed data, group difference was tested by using one-way analysis of variance (ANOVA) followed by a post hoc Bonferroni test for multiple comparisons. For non-normally distributed data, group difference was tested by using Kruskal-Wallis test followed by a Dunn–Bonferroni test for post hoc comparisons. *P*< 0.05*, *P*< 0.01**, *P*< 0.001***. Abbreviations: HC, healthy controls; dACC, dorsal anterior cingulate cortex; GMV, gray matter volume; ReHo, Regional Homogeneity; aITC, anterior part of inferior temporal cortex; pITC, posterior part of inferior temporal cortex; MFC, mid frontal cortex; SFC, superior frontal cortex; rsFC, resting-state functional connectivity; AD, Alzheimer’s disease; aMCI, amnestic mild cognitive impairment; CDR, Clinical Dementia Rating

There were significant differences in GMV of dACC among groups, with significantly lower volumes in different AD groups (mild, moderate and severe group) compared to HC (*p* < 0.05), and lower volumes in severe AD than aMCI (p < 0.05). For both L-aITC and L-pITC, ReHo values were lower in severe AD compared to HC and aMCI groups (p < 0.05). For the FC between the left anterior ITC and left mid frontal cortex, right superior frontal cortex and right angular gyrus, the rs-FC values were lower in severe AD compared to HC and aMCI groups(p < 0.05) (see Fig. 5).

### Correlations between the values extracted from ROI and cognitive function

Pearson correlation analyses were used to examine the relationship between ROI-based neuroimaging variables and clinical cognitive function scores. In HC group, GMV of dACC was significantly negatively correlated with semantic memory(r = − 0.407, *p* = 0.006), but not in the episodic and working memory (see Table S2.). In AD group, GMV of dACC was significantly positively correlated with semantic memory (r = 0.453, *p* = 0.001) and episodic memory (r = 0.334, *p* = 0.015). ReHo values of the left pITC were significantly positively correlated with episodic memory (r = 0.391, *p* = 0.004), and the rs-FC values of the left aITC connected with the right angular gyrus were also significantly positively correlated with episodic memory (r = 0.312, *p* = 0.023). The rs-FC values of the left aITC connect with the left middle frontal cortex were significantly positively correlated with episodic memory (r = 0.338, *p* = 0.013) and semantic memory (r = 0.277, *p* = 0.045) (see Table 4.). There were no significant correlations between ROI-based neuroimaging variables and clinical cognitive function scores in aMCI group (see Table S3.).

**Table 4:** Correlations between ROI-based neuroimaging variables and clinical cognitive function scores in AD group

Cognitive system	Episodic memory		Semantic memory		Working memory	
	r	*p*	r	*p*	r	*p*
^a^GMV of dACC	0.334	0.015*	0.453	0.001**	0.205	0.141
^b^ReHo of left aITC	0.172	0.217	0.049	0.728	0.021	0.884
^b^ReHo of left pITC	0.391	0.004**	0.2	0.151	0.102	0.466
**Left aITC rsFC**^b^						
right angular gyrus	0.312	0.023*	0.082	0.562	0.089	0.525
Left middle frontal cortex	0.338	0.013*	0.277	0.045*	0.084	0.552
right superior frontal cortex	0.108	0.44	−0.035	0.805	−0.128	0.36

The relationship between neuroimaging variables and clinical cognitive function scores were assessed by using Pearson correlation analysis

^a^Adjusted for age, sex, education, and TIV

^b^Adjusted for age, sex, education, and head motion parameters

*P*< 0.05*, *P*< 0.01**, *P*< 0.001***

Abbreviations: *AD* Alzheimer’s disease; *GMV* gray matter volume; *ReHo* Regional Homogeneity; *rsFC* resting-state functional connectivity; *dACC* dorsal anterior cingulate cortex; *aITC* anterior part of inferior temporal cortex; *pITC* posterior part of inferior temporal cortex; *TIV* total intracranial volume; *ROI* region of interest

## Discussion

In this study, we found that highly educated AD patients showed greater gray matter volume atrophy of the dACC, lower ReHo values of left ITC, and decreased FC of the left aITC with the left mid frontal cortex, right superior frontal cortex and right angular gyrus. Meanwhile, we found GMV of dACC shrinked, ReHo values of left ITC decreased, and FC of the left aITC with the left mid frontal cortex, right superior frontal cortex and right angular gyrus decreased with the progression of AD. This suggested brain shrinkage, reduction of ReHo and FC values mean more severe brain damage. As a result, it is reasonable to assume that with the same cognitive function level, individuals with higher cognitive reserve had more severe pathology damage in brain structure and function. These results may seem counterintuitive; at a comparable clinical symptom severity degree, AD neuropathology may be more severe in high educated persons. One probable explanation is that this kind of education background may provide more skills to compensate for the clinical symptoms of the disorder. A series of studies confirmed that biomarkers were more abnormal in higher CR individuals than lower CR individuals at the same degree of cognitive function [7, 11, 14, 37–44]. Whereas, underlying neural mechanism is still unclear. We speculate that intellectual activity may keep neuronal health and enhance self-repair process by stimulating neuronal activity.

Our first finding was that more highly educated AD patients can tolerate more gray matter volume atrophy of the dACC than less educated AD individuals while they have the same degree of cognitive function. This result gives solid evidence for the perspective that higher educated individuals have a relatively higher CR and indicates that ACC may engage in cognitive reserve processes. This gray matter finding is consistent with the previous study which reported that compared to monolinguals, bilinguals have increased gray matter in an extensive cluster along the ACC, starting from its dorsal region and extending towards its ventral region [45]. As one of the brain areas underlying executive control, ACC is more highly stimulated in bilingual speakers and this may result in greater cognitive reserve that compensates for the brain atrophy found in normal aging [46, 47]. Some evidences also showed that bilingualism delays the onset of dementia [48, 49]. Guzmán-Vélez et al. suggested that education and bilingualism may use a similar mechanism to compensate for brain damage, perhaps because a second language is often learned through formal education [50]. That is to say, subjects with a high educational background are more likely to have bilingualism ability. As a result, it is plausible to suppose that early and lifelong education induces beneficial neural changes upon brain structures mediating cognitive control, specifically the ACC. Functionally, the ACC is indirectly related to episodic memory through its influence on related functions such as cognitive control, conflict resolution, motivation, and perseverance [51]. Some researchers raised that cognitive control may be a part of reserve [52]. Previous studies have suggested that ACC is responsible for integrating various input information and regulating the processing in other brain regions [53–55]. We speculate that education induces neural plasticity and provides greater reserve to tolerate greater gray matter volume atrophy [56]. This may also explain why there were no significant correlations observed in HCs and aMCI patients; as education likely improve brain plasticity by developing neural connections rather than increasing gray matter volume in normal cognitive and preclinical AD individuals.

As an important rs-fMRI metric, ReHo has been proposed to be able to effectively quantify synchronization among BOLD time series of a given voxel and its nearest neighboring voxels [57], and could provide valuable spatiotemporal information from a neurobiological perspective [58]. Increasing evidence suggests that ReHo can be used as an imaging biomarker to monitor and/or identify AD pathology and ReHo has also been shown to significantly correlate with cognitive performance [59, 60]. Some experts find that decreases and increases in ReHo values can coexist across multiple brain regions in the patients with the same cognitive diagnosis [61]. This might suggest that it might be too early to conclude an overall effect regarding changes in ReHo value, as dynamics of cerebral blood flow could be highly affected by a wide spectrum of degenerative states and possible interregional reciprocal changes. Our analyses suggested that higher educated AD individuals have relatively lower ReHo values of left ITC. The decrease in ReHo of left ITC in the sever AD group indicated an ongoing profound disconnection process. It is known that in the AD brain, the density of neurofibrillary tangles in the inferior temporal cortex is high and is significantly correlated with dementia severity or neuronal loss [62]. In AD, starting from the inferior temporal gyrus, tau pathology spreads gradually throughout the cortex, resulting in cognitive impairment [63]. The neurofibrillary tangles and tau deposition may lead to decreases in the amplitude and/or phase discordance of BOLD signals within individual neuron clusters. Another study indicated that highly educated prodromal AD patients showed more severe hypometabolism and relative hypermetabolism than individuals with poor education in the left inferior gyri and right superior frontal gyri, respectively [7]. Hypometabolismin the left inferior gyri might be related to low ReHo values. A recent study showed that in the left inferior temporal gyrus, higher levels of education were related to thinner cortical thickness (greater cortical atrophy) [9]. Therefore, we speculate that the left inferior temporal gyrus may play an important role in CR in AD patients.

Besides, we found that higher education was related to the reduced connectivity of the left aITC with the left mid frontal cortex, right superior frontal cortex, and the right angular gyrus in AD. The ongoing profound disconnection process in the left aITC may affect its functional connectivity with the rest of the brain. The left mid frontal cortex is an area known to allocate and coordinate cognitive resources in the working memory [64–67], and is thought to be involved in Chinese reading and reading acquisition [68]. The right angular gyrus is also an area known to be susceptible to AD pathology. The AD-related pathological susceptibility and cognitive related characteristics of these significantly correlated regions in our study indicated that these regions may be components of neural reserve. In summary, our present findings suggested that AD patients with high education can withstand the AD pathology process than those with low levels of education due to neural reserve.

The second finding of this study was that higher educated mild AD patients were better able to withstand greater gray matter volume atrophy, and decreased ReHo values and FC compared with lower educated mild AD patients at the same level of the cognitive performance. Interestingly, the differences in brain structure and function related to education seem to weaken in the more severe AD brains [69]. The result is in line with previous studies that demonstrate the notion that CR mainly occurs at the initial stage of the disease [15, 70, 71]. With the progression of clinical symptoms, the protective impact of education on the brain exits but diminishes. We assume that there exists a fixed critical threshold, at which brain reserve can withstand the brain damage. Once exceeding the threshold, brain reserve capacity will be depleted, leading to specific clinical symptoms. In AD brain, synapses gradually reduce, and the point where synapses reduced to the specific number might be the critical threshold. We therefore suggest that brains with high synaptic density can cope better with AD pathology process before reaching the threshold. There is an abundance of evidence for the threshold effect that higher educated AD individuals can mask dementia manifestations initially, but once going through the threshold, cognition will decline more rapidly [15, 18, 72–75].

Our findings suggest that the protective impact of education mainly occurs in the earlier stages of the AD and a reasonable time-window for therapeutic interventions should therefore be chosen. In mild AD and aMCI, improvement of CR may help resist AD dementia, but when the threshold is reached, CR cannot withstand AD-related brain pathology anymore and intervention will be inefficient. In addition, the aim of most clinical trials is to evaluate the progression of cognitive impairment between drug or intervention group and untreated group. Taking the discrepancy of different level of CR-related cognitive decline into account, clinicians should select different treatment strategies separately for high and low CR in order to improve the curative effect.

In healthy control group, no significant correlations between brain structure and function and education levels were found. One possible reason might be the subject selection, i.e., some subjects with apolipoprotein E4 (APOE4) and others without APOE4. The APOE4 is one of the risk factors for AD [76]. Higher levels of education and higher levels of midlife cognitive activities forestall amyloid deposition by a yet unkown mechanism, which is evident in APOE4 carriers, as APOE4 carriers have more amyloid at the earlier stage and accumulate faster compared with non-APOE4 carriers [77, 78]. Thus, we should consider the effect of APOE4 on cognitively normal subjects. Moreover, ceiling effects [3] and the relatively small sample size may have influenced the result. The MMSE scores in HC group are affected by ceiling effects as many healthy control individuals have the maximum scores of 30. However, cognitive level of these individuals are not equal because their true cognitive state may be significantly higher than 30 [73].

At last, we examined the relation between ROI-based neuroimaging variables and clinical cognitive function scores. In HC group, greater gray matter volume of dACC was associated with poorer semantic memory. This indirectly demonstrated our speculation that education may increase brain reserve by inducing neural plasticity in cognitively healthy elderly individuals. It was an active process. However in AD group, gray matter volume atrophy of dACC was associated with poor semantic memory and episodic memory. We also found that lower ReHo and rs-FC values were associated with poorer semantic memory and episodic memory. This indicates that the later stages of AD dementia is a passive process, the protective role of education on cognition remains but diminishes and patients with higher education can tolerate more severe brain structural and functional damage.

Our study has some limitations. Firstly, the design of this study is a cross sectional study; additional longitudinal studies are necessary to better understand the relationship between brain structure and function characteristics and education. Secondly, our patients were recruited at specialized memory clinics, a multi-center research is necessary to validate the results across different stages of AD. Thirdly, MRI variables we used in this study cannot capture the whole aspect of brain pathology. MRI variables should include different structural measures, such as regional volumes, cortical thickness, measures of white matter integrity, as well as measures of functional networks and molecular markers of pathology. However, it is also true that neuroimaging variables we used here may capture a portion of CR. Fourth, we tested education as a proxy of cognitive reserve but did not evaluate the combined effect of occupation, premorbid IQ, leisure activities, socioeconomic status (SES) or early life linguistic ability. It is difficult to isolate the contribution of formal education to cognitive reserve from that of other variables. People with lower educational levels probably had lower IQ and came from a lower socioeconomic background which is associated with worse nutrition and lack of access to adequate health care, which can also influence brain health. Indeed, it is difficult to disentangle the role of each component of cognitive reserve on shaping the relationship between brain structure and function. Although each aspect might play an independent and additive role, we believe that years of education is a reasonable proxy that reflect the overall consequences of a brain that was not adequately stimulated during childhood. However, it is necessary to measure and account for occupation, SES or other contributors of cognitive reserve instead of only education to offer a more accurate estimate of cognitive reserve for the future research. Finally, subjects with available APOE genotype data were small, so we did not consider the influence of APOE4 on cognitive decline. However, unless APOE genotype is associated with education, it would not have any impact on our findings.

Our study also has obviously strengths. Firstly, without ionizing radiation and invasion, and less expensive than PET, MRI can provide more information about brain properties, so we chose MRI-based biomarkers to explore the correlation between education and AD neuroimaging. Secondly, the integration of brain function and structure holds great promise in improving our understanding of cognition. Indeed, our analysis highlights the importance of considering AD severity in modeling cognitive decline. Thirdly, MRI-based biomarkers are useful to assess the effect of cognitive interventions. Two patients with the same clinical symptoms could differ in MRI-based biomarkers. Understanding the disassociation between cognition and pathology is crucial for successfully preventing AD-related neuropathology and improving our understanding of AD.

## Conclusions

Our results indicate that education is protective against cognitive deterioration, providing support for the CR theory, which predicts that at the same level of clinical symptom severity, higher educated patients can compensate for AD neuropathological manifestations more effectively. Exploring the neural basis of CR might improve our understanding of CR-associated brain structural and functional changes and contribute to targeted intervention. Transcranial direct current or magnetic stimulating the CR-related brain targets might increase CR and slow down the progression of AD.

## Supplementary Information

**Additional file 1: Fig.S1** Results of the cluster-wise multiple regression between years of education and gray matter volume after adjustment for age, sex, MMSE, TIV and Fazekas score(*P*<0.05, cluster-level FWE-corrected). Details of the peaks are given in Table S1. Abbreviations: dACC, dorsal anterior cingulate cortex; L, left; R, right.

**Additional file 2: Fig.S2** Results of the voxel-wise multiple regression between years of education and mean ReHo values after adjustment for age, sex, MMSE, head motion parameters and Fazekas score(*P*<0.05, FDR corrected). Details of the peaks are given in Table S1. Abbreviations: aITC, anterior part of inferior temporal cortex; pITC, posterior part of inferior temporal cortex; L, left; R, right.

**Additional file 3: **Fig**.S3** Results of the voxel-wise multiple regression between years of education and left anterior part of inferior temporal cortex(MNI coordinate: -57,-12,-33) functional connectivity after adjustment for age, sex, MMSE, head motion parameters and Fazekas score(*P*<0.05, FDR corrected). Details of the peaks are given in Table S1. Abbreviations: MFC, mid frontal cortex; SFC, superior frontal cortex; L, left; R, right.

**Additional file 4:.**

## Data Availability

The datasets used and/or analyzed during the current study are available from the corresponding author on reasonable requests.

## Abbreviations

CR: Cognitive reserve
AD: Alzheimer’s disease
aMCI: Amnestic mild cognitive impairment
HC: Cognitively healthy elderly
GMV: Gray matter volume
ReHo: Regional homogeneity
FC: Functional connectivity
dACC: Dorsal anterior cingulate cortex
ITC: Inferior temporal cortex
MMSE: Mini-Mental State Examination
FDG-PET: ^18^F-fluorodeoxyglucose positron emission tomography
CSF: Cerebrospinal fluid
fMRI: Functional MRI
CDR: Clinical Dementia Rating
AVLT: Auditory verbal learning test
VFT: Verbal fluency test
rs-fMRI: Resting-state functional MRI
MNI: Montreal Neurological Institute
FD: Frame-wise displacement
ROI: Regions of interest
TIV: Total intracranial volume
FDR: False discovery rate
aITC: Anterior inferior temporal cortex
