# Supplementary figures and images for: The protective impact of education on brain structure and function in Alzheimer’s disease
Source: BMC Neurol. 2021 Oct 30;21:423. doi: 10.1186/s12883-021-02445-9 (PMC8557004; doi:10.1186/s12883-021-02445-9)

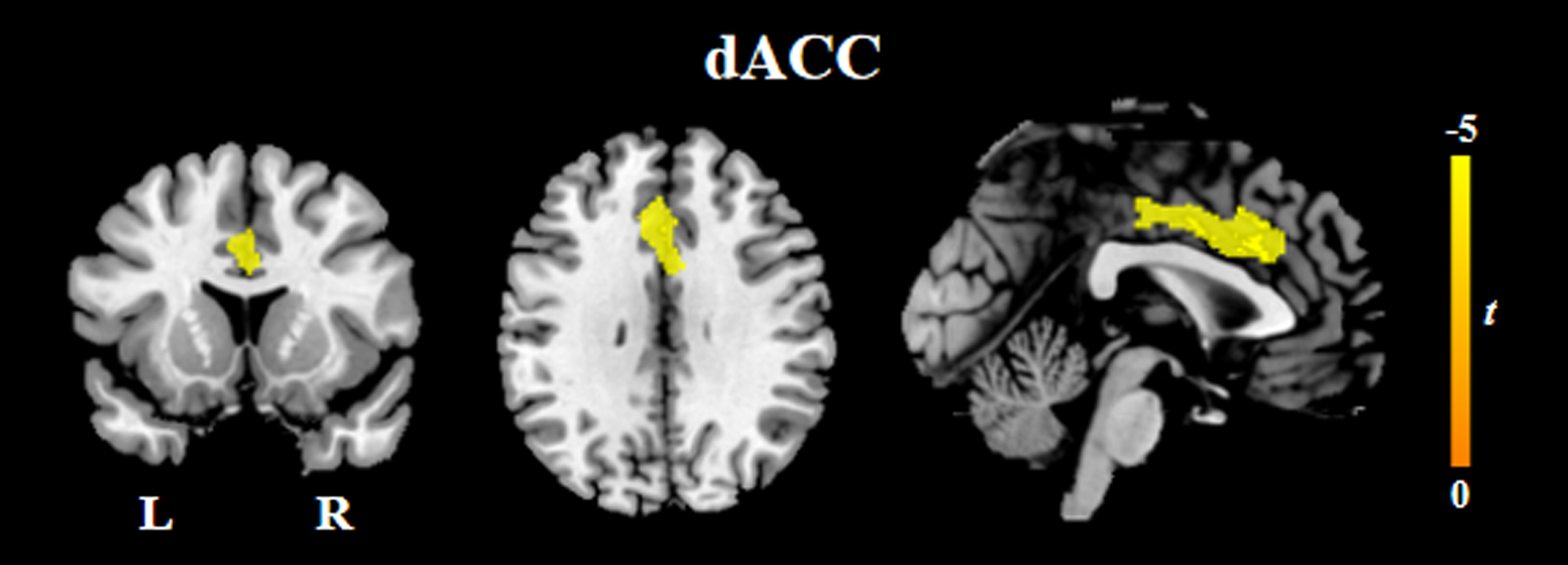

Supplement: Supplementary file 1 — Additional file 1: Fig.S1 Results of the cluster-wise multiple regression between years of education and gray matter volume after adjustment for age, sex, MMSE, TIV and Fazekas score(P<0.05, cluster-level FWE-corrected). Details of the peaks are given in Table S1. Abbreviations: dACC, dorsal anterior cingulate cortex; L, left; R, right. [file 12883_2021_2445_MOESM1_ESM.tif]

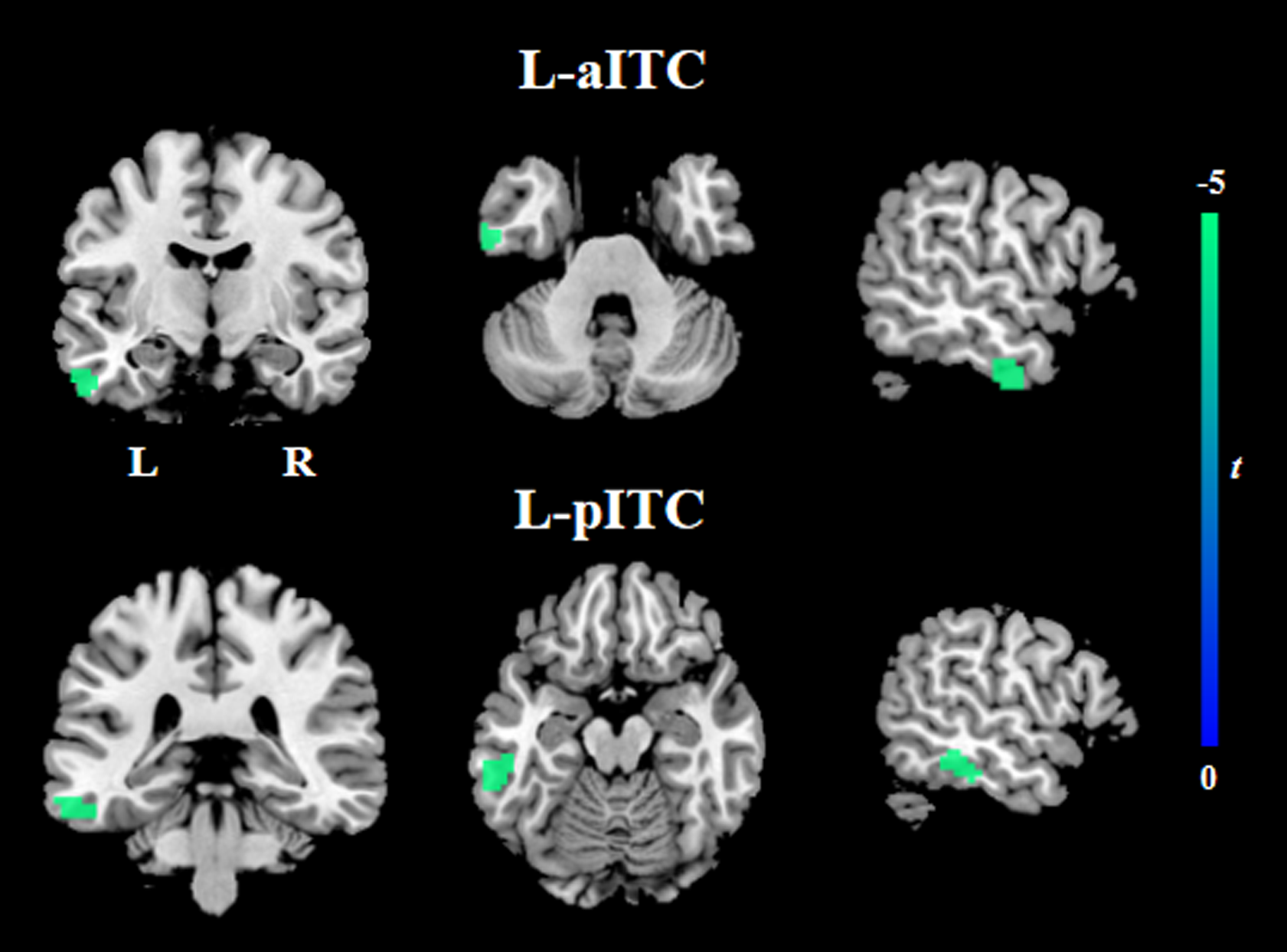

Supplement: Supplementary file 2 — Additional file 2: Fig.S2 Results of the voxel-wise multiple regression between years of education and mean ReHo values after adjustment for age, sex, MMSE, head motion parameters and Fazekas score(P<0.05, FDR corrected). Details of the peaks are given in Table S1. Abbreviations: aITC, anterior part of inferior temporal cortex; pITC, posterior part of inferior temporal cortex; L, left; R, right. [file 12883_2021_2445_MOESM2_ESM.tif]

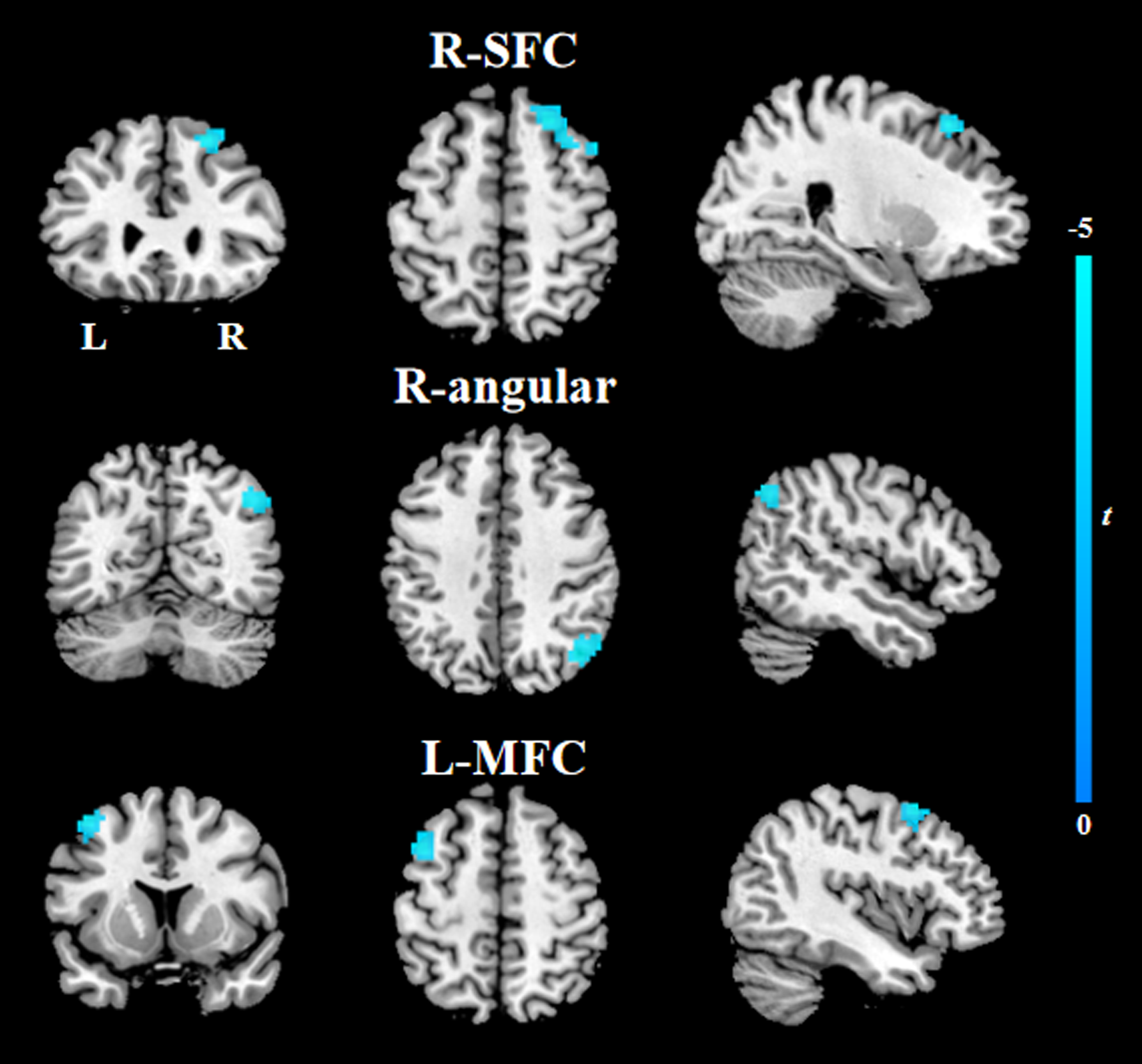

Supplement: Supplementary file 3 — Additional file 3: Fig.S3 Results of the voxel-wise multiple regression between years of education and left anterior part of inferior temporal cortex(MNI coordinate: -57,-12,-33) functional connectivity after adjustment for age, sex, MMSE, head motion parameters and Fazekas score(P<0.05, FDR corrected). Details of the peaks are given in Table S1. Abbreviations: MFC, mid frontal cortex; SFC, superior frontal cortex; L, left; R, right. [file 12883_2021_2445_MOESM3_ESM.tif]
