# Supplementary material for: The protective impact of education on brain structure and function in Alzheimer’s disease
Source: BMC Neurol. 2021 Oct 30;21:423. doi: 10.1186/s12883-021-02445-9 (PMC8557004; doi:10.1186/s12883-021-02445-9)
Supplement: Supplementary file 4 — Additional file 4:. [file 12883_2021_2445_MOESM4_ESM.docx]

**Supplemental material**

TableS1

Results of voxel/cluster-based regression analyses between neuroimaging parameters and years of education

| Region | Number of voxels | *t*-score | MNI coordinates | | |
| --- | --- | --- | --- | --- | --- |
|  |  |  | x | y | z |
| **Negative correlations between years of education and GMV** | | | | | |
| dorsal anterior cingulate cortex | 1732 | -5.06 | 0 | 15 | 34 |
| **Negative correlations between years of education and ReHo** | | | | | |
| left anterior part of inferior temporal cortex | 40 | -6.01 | -57 | -12 | -33 |
| left posterior part of inferior temporal cortex | 78 | -4.82 | -63 | -36 | -15 |
| **Negative correlations between years of education and left aITC rsFC** | | | | | |
| right angular gyrus | 61 | -4.79 | 45 | -66 | 42 |
| left mid frontal cortex | 60 | -5.81 | -39 | 15 | 57 |
| right superior frontal cortex | 77 | -5.17 | 24 | 27 | 54 |

Structural MR (GMV): Adjusted for age, sex, MMSE, TIV and Fazekas score. The region listed is statistically significant at *p*＜0.05 cluster**-**level (FWE corrected).

Resting-state fMRI (ReHo and rsFC): Adjusted for age, sex, MMSE, head motion parameters and Fazekas score. All regions listed are statistically significant at *p*＜0.05 voxel**-**level (FDR corrected).

Coordinates (x,y,z) are given in MNI standard space.

Abbreviations: GMV, gray matter volume; ReHo, Regional Homogeneity; rsFC, resting-state functional connectivity; MNI, Montreal Neurological Institute; aITC, anterior part of inferior temporal cortex; MMSE, Mini-Mental State Examination; TIV, total intracranial volume.

Table S2

Correlations between ROI-based neuroimaging variables and clinical cognitive function scores in HC group

| Cognitive system | Episodic memory | | Semantic memory | | Working memory | |
| --- | --- | --- | --- | --- | --- | --- |
|  | r | *p* | r | *p* | r | *p* |
| ^a^GMV of dACC | -0.237 | 0.122 | -0.407 | 0.006** | 0.107 | 0.488 |
| ^b^ReHo of left aITC | -0.089 | 0.564 | -0.152 | 0.326 | -0.06 | 0.7 |
| ^b^ReHo of left pITC | -0.241 | 0.114 | -0.039 | 0.801 | 0.13 | 0.399 |
| **Left aITC rsFC**^b^ |  |  |  |  |  |  |
| right angular gyrus | 0.039 | 0.802 | 0.014 | 0.929 | 0.132 | 0.393 |
| Left middle frontal cortex | 0.109 | 0.479 | 0.088 | 0.571 | 0.027 | 0.862 |
| right superior frontal cortex | 0.03 | 0.848 | -0.065 | 0.674 | 0.052 | 0.738 |

The relationship between neuroimaging variables and clinical cognitive function scores were assessed by using Pearson correlation analysis.

^a^Adjusted for age, sex, education, and TIV.

^b^Adjusted for age, sex, education, and head motion parameters.

*P*＜ 0.05*, *P*＜ 0.01**, *P*＜ 0.001***

Abbreviations: HC, healthy control; GMV, gray matter volume; ReHo, Regional Homogeneity; rsFC, resting-state functional connectivity; dACC, dorsal anterior cingulate cortex; aITC, anterior part of inferior temporal cortex; pITC, posterior part of inferior temporal cortex; TIV, total intracranial volume; ROI, region of interest.

Table S3

Correlations between ROI-based neuroimaging variables and clinical cognitive function scores in aMCI group

| Cognitive system | Episodic memory | | Semantic memory | | Working memory | |
| --- | --- | --- | --- | --- | --- | --- |
|  | r | *p* | r | *p* | r | *p* |
| ^a^GMV of dACC | 0.138 | 0.323 | 0.08 | 0.569 | 0.259 | 0.061 |
| ^b^ReHo of left aITC | -0.043 | 0.758 | -0.106 | 0.449 | 0.006 | 0.968 |
| ^b^ReHo of left pITC | -0.132 | 0.345 | 0.038 | 0.785 | -0.087 | 0.537 |
| **Left aITC rsFC**^b^ |  |  |  |  |  |  |
| right angular gyrus | -0.139 | 0.321 | 0.05 | 0.724 | -0.062 | 0.658 |
| Left middle frontal cortex | -0.259 | 0.061 | 0.016 | 0.91 | -0.184 | 0.186 |
| right superior frontal cortex | 0.026 | 0.856 | -0.156 | 0.266 | -0.099 | 0.483 |

The relationship between neuroimaging variables and clinical cognitive function scores were assessed by using Pearson correlation analysis.

^a^Adjusted for age, sex, education, and TIV.

^b^Adjusted for age, sex, education, and head motion parameters.

*P*＜ 0.05*, *P*＜ 0.01**, *P*＜ 0.001***

Abbreviations: aMCI, amnestic mild cognitive impairment; GMV, gray matter volume; ReHo, Regional Homogeneity; rsFC, resting-state functional connectivity; dACC, dorsal anterior cingulate cortex; aITC, anterior part of inferior temporal cortex; pITC, posterior part of inferior temporal cortex; TIV, total intracranial volume; ROI, region of interest.

**Figure legends**

**Fig.S1 Results of the cluster-wise multiple regression between years of education and gray matter volume after adjustment for age, sex, MMSE, TIV** **and Fazekas score(*P*＜0.05, cluster-level FWE****-corrected).** Details of the peaks are given in Table S1. Abbreviations: dACC, dorsal anterior cingulate cortex; L, left; R, right.

**Fig.S2 Results of the voxel-wise multiple regression between years of education and mean ReHo values after adjustment for age, sex, MMSE, head motion parameters and Fazekas score(*P*＜0.05, FDR corrected).** Details of the peaks are given in Table S1. Abbreviations: aITC, anterior part of inferior temporal cortex; pITC, posterior part of inferior temporal cortex; L, left; R, right.

**Fig.S3 Results of the voxel-wise multiple regression between years of education and left anterior part of inferior temporal cortex(MNI coordinate: -57,-12,-33) functional connectivity after adjustment for age, sex, MMSE, head motion parameters and Fazekas score(*P*＜0.05, FDR corrected).** Details of the peaks are given in Table S1. Abbreviations: MFC, mid frontal cortex; SFC, superior frontal cortex; L, left; R, right.
